# Are Lay People Able to Estimate Breeding Bird Diversity?

**DOI:** 10.3390/ani12223095

**Published:** 2022-11-10

**Authors:** Janina Vanhöfen, Nick Schöffski, Talia Härtel, Christoph Randler

**Affiliations:** Didactics of Biology, Department of Biology, Eberhard-Karls-University Tübingen, Auf der Morgenstelle 24, D-72076 Tuebingen, Germany

**Keywords:** biodiversity, citizen science, green space, ornithologist, species richness

## Abstract

**Simple Summary:**

The experience of nature is different for everybody. Some studies used perceived biodiversity as an approach to measure the biodiversity of a location. This present study assessed if laypeople could estimate bird species richness at specific places in southwest Germany. We compared their assessment with bird counts of professional ornithologists and data from the citizen science platform Ornitho (Germany). With a survey, laypeople were asked about the bird diversity they perceived at a given place. About 30 people were surveyed per place, summing up a total of 1184 respondents. The results show that laypeople have a generally good assessment of the bird species richness in recreational areas, correlating with the data of professionals. But since the surveys often were done in the afternoon and their assessment coincided with the professional data of morning and total counts, laypeople seem to make their assessment of biodiversity due to other factors than actual birds seen. The counts of professional ornithologists and citizen science data did not correlate; therefore, we suggest carrying out own professional surveys if possible.

**Abstract:**

Studies about biodiversity and well-being used different approaches to assess biodiversity, e.g., scientific counts and censuses or perceived biodiversity estimated by the respondents. Here, we assessed whether laypeople could estimate the breeding bird diversity or species richness at specific places. For comparison, we carried out bird censuses with standard methods of professional ornithologists and used citizen science data from the internet platform Ornitho (Germany). Lay people from the public (1184 respondents) were surveyed between May and July 2022 at 40 different places in southwest Germany between Rottenburg/Tübingen and Stuttgart following the catchment of the river Neckar (30 people surveyed per place). People were asked to estimate the bird species richness/diversity at this current place. Here, we show that the data from the citizen science platform does not correlate with the professional census counts nor with the perceived species richness of laypeople. Laypeople have a generally good assessment of the bird species richness, correlating with the data of professionals (r = 0.325, *p* = 0.041). On average, the number of species assessed by laypeople lies in between the values of the professional morning and afternoon census. People were most often surveyed in the afternoon; therefore, their assessment must be done on other factors than actual birds present. This result is valuable for future studies on the connection between biodiversity and well-being.

## 1. Introduction

As soon as people go outside, they experience nature and biodiversity, even in small urban green spaces [1,2]. Being in contact with nature and biodiversity helps people to restore psychologically, additionally to widely being recognized for having positive effects on human health and well-being [2,3,4,5,6]. Psychological restorativeness means replenishing cognitive resources and reducing stress [4,6]. The attention restoration theory (ART) states that spending time in nature allows recovery from attention fatigue [4,5]. Meanwhile, the stress reduction theory (SRT) proposes that a high biodiversity level benefits psychophysiological stress recovery [6]. Moreover, the specific knowledge of people is not related to beneficial rewards from nature [7]; thus, people at all levels of biodiversity knowledge may benefit from being in nature.

Evaluation of biodiversity may depend on the condition of laypeople and not on their knowledge. Although the benefit of biodiversity on psychological restoration, well-being, and positive emotions is unquestioned, it is still unclear which specific measures of diversity may be responsible for this relationship. Studies about biodiversity and well-being used different approaches to assess biodiversity, e.g., real counts or censuses [8], perceived biodiversity estimated by the respondents [3] or even virtual experience of green spaces [9]. But different studies revealed inconsistencies in the relationship between actual biodiversity (measured as species richness), perceived biodiversity, and well-being. Some studies found psychological benefits with higher actual biodiversity [8,10]. In contrast, there was a strong positive relationship between well-being and perceived biodiversity in some studies. Fisher, et al. [3] showed that if sites were perceived as species-rich, they were perceived as more restorative, resulting in the improved well-being of citizens. Nghiem, et al. [11] assessed perceived biodiversity and found that perceived animal diversity contributes to perceived (psychological) restorativeness. In general, perceived biodiversity has been more consistently related positively to well-being, highlighting the importance of psychological benefits.

Moreover, some studies took the biodiversity knowledge of the respondents into account when assessing the value of nature [12]. However, many people are unfamiliar with the biodiversity in their environment [13]. Most people have inaccurate ideas about the number of species in their country or worldwide. For example, in Switzerland, plant species richness was strongly overestimated [14]. Even though laypeople appreciate species richness, they do not seem to be able to accurately perceive it in their local green space. Dallimer, et al. [8] found that there was no consistent association between the actual and perceived species richness of plants, birds, and butterflies. The degree of mismatch even grew as the participants’ species knowledge declined. Shwartz, et al. [15] concluded that people underestimate species richness and that there is no correlation between observed and estimated species richness. Other studies found no relationship between bird diversity and perception by park visitors. Visitors underestimated avian diversity in city parks, and public knowledge did not relate to species richness [16]. In contrast, Fisher, et al. [3] found that participants accurately estimated bird species richness around them and perceived sites with greater proportions of vegetation and water as more natural. Fuller, et al. [17] identified that participants could detect plant species richness fairly accurately.

Many studies concerning biodiversity and the effect on people used birds as a proxy for actual biodiversity [8,16,18,19,20,21,22]. In addition, other than being used in previous studies, birds are used for various reasons, for example, their capacity to draw attention and fascinate due to color, movement, or song [23]. Additionally, birds are generally preferred by laypeople and more easily distinguished as different species compared to other taxonomic groups, for example, insects [24]. Therefore, we used the estimate of bird diversity as an estimate for biodiversity to compare the assessments of laypeople, professional ornithologists, and citizen science data.

Citizen Science (CS) makes it possible to involve anyone with curiosity and access to the internet to contribute to scientific questions and databases. This facilitates the collection and distribution of all kinds of taxonomic data with a wide geographic distribution at minimal cost and could even possibly replace museums’ roles in the future for supplying a biodiversity baseline [25]. With regards to birdwatching, there is a large [birding] community, and contributions to CS are manifold. Birdwatchers are a quite heterogeneous group [26], ranging from novices up to professionals. Randler [27] showed that users of the CS platform Ornitho showed higher skill and competence, more investment, and higher personal commitment to birdwatching than birdwatchers not using the CS platform. This suggests that the data quality of such platforms is comparatively high. Most lists of CS data have also been found to be mostly complete at the family level when compared to professional data and provide prompt records for new emerging species [28]. Since most species are correctly identified as well [29], this means CS data can be a good predictor for species richness [20]. Robinson, et al. [30] showed a count difference biased toward undercounts of contributors to the CS platform eBird compared to a professional ornithologist. They also concluded that species misidentification was unlikely to be an important factor in the counting errors.

We here compare bird diversity among professional ornithologist counts, CS data, and laypeople assessment. For the CS data, we used the large German platform Ornitho and aimed to check if the results of this platform were consistent with the professional census and could be used for our surveys. Many professional ornithologists also contribute to many different CS platforms, making these possibly easy solutions for scientific questions that are difficult to solve for one or two professionals alone.

Since the ability of laypeople to detect the actual level of species richness in an area varies on an individual level, it is important to know to which degree laypeople can accurately estimate the bird diversity at a given place. This knowledge not only translates to the well-being of people but also to their connectedness to nature. If laypeople can estimate species richness, this knowledge can be used for future environmental measures and activities. Therefore we here asked the questions: (1) whether laypeople could correctly estimate actual occurring species numbers of birds in recreational areas and (2) whether the results of CS projects match with professional bird censusing of breeding birds. Further, we study this question on the individual level of the respondents and on the place level, following the concept of swarm intelligence [31].

## 2. Measurements and Methods

### 2.1. Study Area

We conducted the study at 40 locations between Rottenburg am Neckar and Stuttgart along the Neckar River in southwest Germany. The places differ in degree of urbanization and include blue spaces as well as green spaces for a well-rounded picture of the diversity this area has to offer. All of them are recreational areas that are easily accessible for people, either by foot, car, or public transportation.

### 2.2. Professional Bird Censuses

Bird species richness was surveyed at each site by trained ornithologists (authors NS, CR). Using standard protocols [32], bird counts were carried out two times in the morning and once in the afternoon, with each morning survey having at least three weeks in between. In a 250 m radius on footpaths, which visitors also experience, all bird species and individuals seen and heard in a time span of 15 min were recorded. Since estimated bird densities increase with time [33], we expect this method to cover most of the bird species available in these locations. Data were collected between March and June 2022. We compiled three species lists per location of these bird surveys: professional morning count, professional afternoon count, and professional total count. The professional total count compiled all encountered bird species of all recordings at one location; thus, each species contributed once to the dataset.

### 2.3. Citizen Science—Ornitho Data

Approximately 33,000 people use the German-based birding platform Ornitho (www.ornitho.de, accessed on 10 July 2022) to submit bird observations to CS projects. Ornitho comprises the countries Germany, Switzerland, Austria, Lichtenstein, and Luxemburg but is also widely used in other parts of Europe. The data are incorporated into a lot of different scientific and conservational evaluations, making it a valuable dataset of bird species of central Europe. Our CS Data was compiled following Ding, Xiong, Ji, Lu, Zhu and Huang [28]. The Ornitho website featured data from 31 out of our 40 locations, which has been kindly provided to us by the Ornithologische Gesellschaft Baden-Württemberg (OGBW). We used the data from March to June of 2022, with permission of the Steuerungsgruppe of the OGBW. The number of bird species was compiled into one count per place, summing up all species recorded. The sampling periods between the professional count and the CS were identical to guarantee a fair comparison.

### 2.4. Lay People Surveys

A survey study was carried out with pedestrians in all 40 study areas. The surveys were carried out by a Ph.D. student (TH) and some Master’s students at the University of Tübingen. Master students received course credits. People over the age of 18 were recruited to participate in the questionnaire if they had already been in the respective area for a while. They were asked to participate in the study and given the questionnaire on paper with a ballpoint pen as a participation gift. In total, 1184 people participated in the survey. The study has been granted ethical permission by the ethics committee of the Faculty of Social Sciences and Economics of the University of Tübingen.

The questionnaire included 24 questions about location and biodiversity parameters and emotional and well-being items. The question of estimated bird species richness at the respective place was based on a categorical scale containing nine categories (0–5; 6–10; 11–20; 21–30; 31–40; 41–50; 51–60; 61–70; over 70 species present; scale adapted after Ferraro, et al. [34] and Southon, et al. [35]). We also asked about birding specialization based on how many bird species participants can recognize by appearance without help from books or apps [36]. In addition, some demographic variables were asked for: age in years, gender (male, female, diverse), and graduation (university degree, coded dichotomous “yes or no” with “yes” meaning at least a bachelor’s degree).

### 2.5. Statistical Analysis

Due to the structure of the data sets, we did a nonparametric correlation analysis with Spearman-rho as the correlation coefficient and a two-sided significance testing. Additionally, we did a Wilcoxon test to compare the categories within the same places. Demographic differences were assessed using Mann–Whitney U tests. For statistical analysis, the program SPSS (Version 28.0.0.0) was used.

We used the following variables: The number of species in total from three census counts (two mornings, one afternoon) = professional total count; the number of species from two census counts in the morning (morning bird diversity) = professional morning count; the number of species from census counts in the afternoon = professional afternoon count; the total number of species in ornitho.de for March to July 2022 = Ornitho. As the data were collected in absolute numbers for the professional count and in categories for the pedestrian assessment (see above), we also transformed the precise counts into categories to make the data comparable for the Wilcoxon tests. Since we had only one item per professional count and CS data, we first analyzed the laypeople’s assessment on the place level, summarizing all their assessments together as a mean score per place based on the concept of swarm intelligence [31]. Thus, we considered the 30 respondents per place as an aggregated expert. After that, we checked if the results kept consistent when analyzed on an individual level.

As demographic effects may have an influence on bird species knowledge [36], we analyzed the estimated species richness with respect to age, gender, self-reported birding skill level, and university degree.

## 3. Results

The professional morning count strongly correlated with the professional afternoon count (r = 0.396, *p* = 0.011) and the professional total count (r = 0.960, *p* < 0.001), making the surveys of the ornithologists inherently consistent, and the morning and the total count nearly identical. The professional afternoon count and the professional total count also correlated significantly (r = 0.525, *p* < 0.001).

The CS data provided by Ornitho did not correlate with any of the professional censuses (Professional Morning Count: r = 0.278, *p* = 0.131; Professional Afternoon Count: r = 0.165, *p* = 0.375; Professional Total Count r = 0.307, *p* = 0.093).

The survey had a slight surplus of women (1184 participants: 54.9% female and 44.1% male and participants, 0.2% identified as diverse, 0.8% did not answer). Over half (55.7%) of the participants reported a university degree. The survey covered a broad age range, with the age of participants ranging from 18 to 89 years old (1.9% did not answer the question of age). The mean age was 43.34 years, with an SD of 17.75 years.

Age correlated negatively with the estimated number of species (r = −0.076, *p* = 0.011). A Mann–Whitney U-test showed a slightly significant difference among gender (Z = −1.996, *p* = 0.046), with males estimating species richness slightly higher. University degree had no impact on estimated species richness (Mann–Whitney U-Test Z = −0.803, *p* = 0.422), but self-reported bird knowledge significantly correlated positively with the estimated number of species (r = 0.102, *p* < 0.001). Thus, higher skilled pedestrians estimated the species richness higher. The perceived species richness of laypeople correlated weakly with the professional total count (r_s_ = 0.085, *p* = 0.004) but not with the CS data (r_s_ = −0.020, *p* = 0.551).

**Figure 1:**
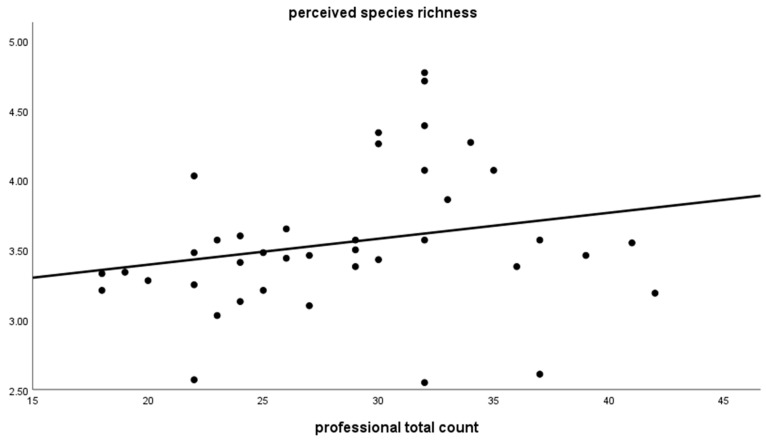
Perceived species richness of laypeople (measured in categories, averaged per place) correlated with the professional total count (measured in absolute numbers).

Aggregated on the place level, the perceived species richness of laypeople correlated with the professional total count (Table 1; Figure 1) even when they were recoded into the same categories, but it did not correlate with the CS data. However, the correlation coefficient was higher in the aggregated data based on the place level (r_s_ = 0.3) following the approach of swarm intelligence compared with the individual level correlation (r_s_ = 0.085).

**Figure 2:**
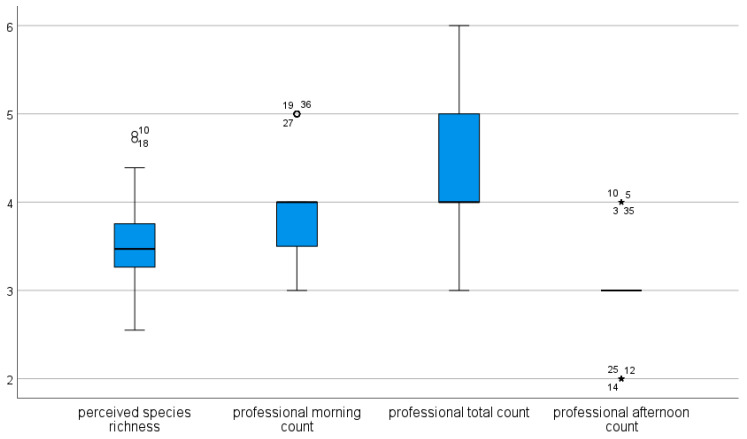
Boxplot of the results of the Wilcoxon test of the recoded variables into the same categories as the laypeople’s assessment: 1 = 0–5; 2 = 6–10; 3 = 11–20; 4 = 21–30; 5 = 31–40; 6 = 41–50; 7 = 51–60; 8 = 61–70; 9 = over 70. Professional total count: Z = −5.028, *p* < 0.001 (negative ranks); professional morning count: Z = −3.166; *p* = 0.002 (negative ranks); professional afternoon count: Z = −4.382, *p* < 0.001 (positive ranks). The circles indicate outliers (numbers are respective case numbers), the * indicate extreme outliers. Numbers represent the places.

Using the converted variables (absolute bird species richness recoded in similar categories as the perceived species richness estimates), a Wilcoxon test of the professional counts with the perceived species richness of laypeople (place level) was done as a follow-up (Figure 2). This analysis showed that laypeople’s assessment in categorical terms is lower compared to the professional morning or total count but higher than the professional afternoon count. This suggests that the laypeople underestimated the morning but overestimated the afternoon species richness.

**Figure 3:**
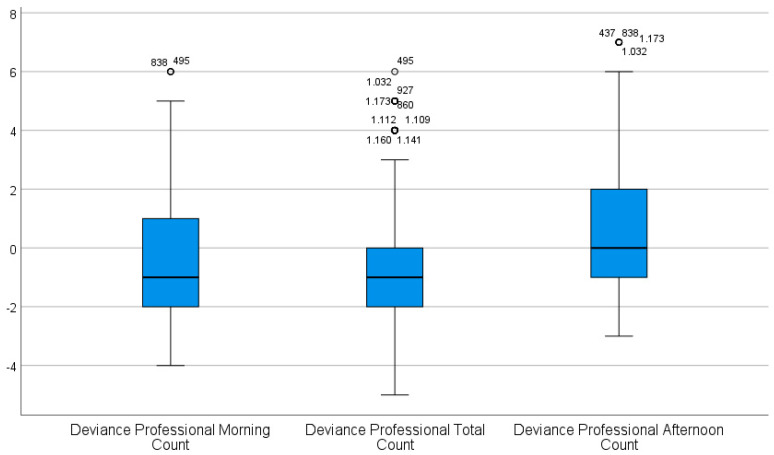
Boxplot of the deviance of the species richness estimates of the people from the professional counts. On the individual level, respondents underestimated the morning and total count by about one category. The circles indicate outliers (the numbers are the respective case numbers).

To check this assumption further, we subtracted the perceived species richness on an individual level from the categorized professional counts. The result confirms the hypothesis that people underestimate professional total and morning count. Figure 3 shows the deviance of the species richness estimates of the people from the professional counts. On the individual level, respondents underestimated the morning and total count by about one category (Figure 3).

## 4. Discussion

This study compared bird diversity among professional ornithologist counts, CS data, and laypeople’s assessment of perceived species richness. We asked whether the results of the CS projects match with professional bird censusing and whether laypeople could correctly estimate the actual numbers of birds in recreational areas.

### 4.1. Professional Counts

Even though the professional afternoon count showed lower values, it is strongly correlated with the other two counts. Looking at the species numbers, the afternoon count does not add much to the total. Total and morning species richness are nearly identical, meaning that the professional morning count could be sufficient in future study designs. Even though afternoon bird abundances were previously positively associated with a lower prevalence of depression, anxiety, and stress [37], fewer birds are present to experience during this time of day. However, it is also the time of day when people experience nature most. Depending on the study design, morning counts at the peak of the breeding season may be suitable, but afternoon counts may provide additional data for specific research questions [37], especially when social questions and human dimensions are in the focus of a study.

The method used in the professional ornithologist’s assessment of bird species is useful because it is easy to apply and provides consistent results. Since estimated bird densities increase with time [33], this method should cover most species. Other studies also used a comparable amount of time but walked transects off trails [19]. This was considered not useful for the present study since we wanted to compare our data with laypeople’s assessments, and the pedestrians stayed on walkways. Since all counts were carried out in the same time span from March to July, most breeding birds in southwest Germany should be covered. The assessments of professional ornithologists are also consistent with each other.

### 4.2. Citizen Science

The CS data are best compared with the professional total count since both contain multiple counts independent of the time of day. Still, the data from the platform Ornitho did not correlate with any of the professional data. Across platforms, the species checklists of different sources do often have considerable discrepancies, even among expert-curated lists, although CS data seem to be mostly complete at the family level when compared to professional data [28]. Even though this is a surprise, it can be sufficiently explained by the differences in data acquisition. The vast majority of data available at Ornitho was collected by the observers during their field trips by chance. As a rule, the data are not the result of systematic studies and are made by an array of observants of different skill levels. Callaghan, et al. [38] showed that CS data are usually biased towards larger and more common species, which are mostly correctly identified [29]. Therefore, for specific scientific questions, CS data may be sufficient. Depending on the research objective, a professional and, most importantly, a systematic count may still be warranted. However, as a cautionary note, our data failed the significance marginally, meaning that a higher sample size may have produced a significant correlation between the CS data and the professional total counts. Therefore, it may be useful relying on CS data, especially when own counts are not feasible.

### 4.3. Lay People’s Estimation of Species Richness

Hooykaas, et al. [13] found that laypeople’s species knowledge increased with age and educational level. Further, Randler and Heil [36] showed that older people scored higher in bird species knowledge tests, meaning that older people can probably better estimate the number of species present at the moment. In our study, age correlated negatively with the perceived species richness estimates, meaning that younger people estimate the species diversity higher than older ones. However, both Hooykaas, et al. [13] and Randler, et al. [39], as well as Randler and Heil [36], explicitly applied a test of species knowledge, while in this current study, we asked the pedestrians for their species richness estimates. This may explain the differing results.

Meanwhile, gender was slightly related to the estimated number of species, with men tending to have a higher assessment of the bird species richness. This is in line with other studies concluding that men are more interested in birding [26,40]. Graduation or university degree did not impact the estimated species count, which is not surprising since everyone can experience nature independently of education.

Self-reported bird knowledge strongly correlated positively with the estimated number of species, meaning that people who think of themselves as having more knowledge of birds make a higher estimate of the occurring bird diversity. Dallimer, et al. [8] could also show that people with higher identification skills are more likely to perceive species richness accurately. But can self-assessments of knowledge be considered a reliable measure? Especially recent studies clearly showed that the self-assessment of bird species knowledge measured by birding specialization (encompassing visual competence, acoustic competence, and self-assessment from novice to expert) based on Lee and Scott [41] correlated with scores from a knowledge test (about 0.7; [36,42]). These studies suggest that using a simple self-classification item rather than a detailed test may make such surveys easier and more convenient.

Our study showed that laypeople could roughly estimate bird species richness in relative terms. Their assessment correlated with the professional morning count and professional total count. Even though they generally underestimated species richness, they did this in a consistent manner. This coincides with Fisher, et al. [3], who found that participants could accurately estimate bird species richness around them. Participants in this study generally assessed the bird diversity lower than the professional morning and total census. This difference probably exists because of the higher number of counts provided by professional ornithologists. People were surveyed only once, while the professional total count consisted of three censuses. Moreover, professional ornithologists went out to specifically look for birds; meanwhile, laypeople did not. They mostly went for relaxation purposes. The pedestrian surveys were mostly done in the afternoon, but participants estimated bird diversity higher than the actual afternoon count and lower than the morning and total count. If people went outside looking specifically for birds, their assessment could possibly be even closer to the professional counts.

As people seem to be able to relatively assess total species richness at a time of day when it is not fully experienceable, they may base their assessment on other cues, such as tree cover or perceived diversity of the area in general, rather than on actual bird diversity. Perceived richness was previously positively associated with vegetation height, evenness, and colorfulness, suggesting that these are cues for estimating species richness [35]. In a study in South America, people estimated bird diversity as lower because they perceived only a small fraction of the overall diversity. Their awareness is especially biased toward the most abundant species [16]. Accordingly, people seem to be biased in their perception of biodiversity, looking only at what grabs their attention. Ishibashi, et al. [43] suggest that both the individual´s personal characteristics and the species’ ecological traits determine park users’ recognition of local biodiversity. Other studies indicate that residents’ valuations of ecosystem services are linked to their perceptions of bird species richness rather than the actual species richness. People may perceive only a subset of the birds in their neighborhoods [44]. Cameron, et al. [19] found that avian biodiversity was closely correlated with the participant’s perception of biodiversity, even though participants rated biodiversity as the number of types of plants/trees/animals in general. People probably perceive biodiversity as high or low, independent of their specific knowledge of birds.

According to our studies, laypeople’s assessment of bird diversity coincides with professional counts, meaning it can be used for further studies. Since biodiversity and well-being are linked, and access to public green spaces and bird species richness are expected to decline in the future [45], it is more important than ever to find ways to preserve the link between people´s perception, experience, and biodiversity.

## 5. Conclusions

The assessment of bird species diversity by professional ornithologists and CS Data did not correlate, meaning professional biodiversity censuses are not replaceable. Therefore, we suggest conducting professional surveys for studies based on bird diversity and well-being. Even though CS data may not be useful for biodiversity assessments as in this study, they could still be useful to assess more large-scale distribution of birds as well as monitoring of alien-species and rare species.

Lay people can roughly estimate the species richness of birds in recreational areas. But since they often went to the area in the afternoon, but their assessment coincided with the professional data of morning and total counts on the aggregated place level, laypeople seem to make that assessment due to other factors than actual birds seen. Therefore, their assessment of biodiversity is subjective and useful for studies on perception, well-being, and others, but not so much as a replacement for actual biodiversity counts. Therefore, it is important to know how laypeople perceive their surroundings when studying in this area.

## Figures and Tables

**Table 1:** Spearman-Rho correlation analysis results of the different bird count sources with the estimation of species present by laypeople. We repeated the analysis with the variables (absolute species richness) converted into the same categories as the laypeople’s assessment: 1 = 0–5; 2 = 6–10; 3 = 11–20; 4 = 21–30; 5 = 31–40; 6 = 41–50; 7 = 51–60; 8 = 61–70; 9 = over 70.

Measured Species Richness (Absolute and in Corresponding Categories)	Perceived Species Richness
Professional Morning Count (absolute)	0.353 *
Professional Morning Count (category)	0.297
Professional Afternoon Count (absolute)	0.158
Professional Afternoon Count (category)	0.098
Professional Total Count (absolute)	0.325 *
Professional Total Count (category)	0.312 *
Ornitho (absolute)	−0.126
Ornitho (category)	−0.097

* *p* < 0.05.

## Data Availability

On request from authors.
